# Enrolling Persons with Dementia in Hospice: A Qualitative Synthesis of Caregivers’ Experiences

**DOI:** 10.1093/geroni/igaf122.2578

**Published:** 2025-12-31

**Authors:** Oonjee Oh, Connie Ulrich, Lauren Massimo, George Demiris

**Affiliations:** University of Pennsylvania, Philadelphia, Pennsylvania, United States; University of Pennsylvania School of Nursing - Philadelphia, PA, philadelphia, Pennsylvania, United States; University of Pennsylvania, Philadelphia, Pennsylvania, United States; University of Pennsylvania, Philadelphia, Pennsylvania, United States

## Abstract

Hospice is a specialized palliative care geared towards the end of life, aiming to relieve the suffering of terminally ill individuals and their families. While persons with dementia (PwD) have high palliative care needs near death, they often receive suboptimal end-of-life care and experience challenges enrolling in hospice. Due to their progressive loss of cognitive abilities, caregivers are heavily involved in end-of-life decision-making and care transitions on behalf of PwD. Such intense involvement suggests that caregivers are in a position to offer valuable insights regarding the provision of quality end-of-life care for PwD. Yet, their experiences throughout hospice entry have not been comprehensively explored. Therefore, we conducted a systematic review of qualitative evidence that describes caregivers’ experience enrolling a loved one with dementia in hospice. Based on a literature search from 5 major bibliographic databases, 22 studies were included. We conducted a thematic synthesis and identified 5 themes: 1) caregivers’ perception towards late-stage dementia, 2) whether hospice is the right choice for PwD, 3) caregiving burden, 4) perception towards their loved one’s death and hospice, and 5) information and hospice access. From these themes, we identified 15 barriers and 11 facilitators. There was a significant gap between the needs of caregivers caring for a seriously ill loved one with dementia and the circumstances under which resources like hospice could be offered. Current hospice referral processes and policies may not adequately reflect the complexity of severe dementia and the unique needs of caregivers at the intersection of dementia and end-of-life care.

